# Supplementary material for: Ubiquilin Networking in Cancers
Source: Cancers (Basel). 2020 Jun 15;12(6):1586. doi: 10.3390/cancers12061586 (PMC7352256; doi:10.3390/cancers12061586)
Supplement: Supplementary file 1 [file cancers-12-01586-s001.pdf]

# Supplementary Materials: The Ubiquilin Networking in Cancers

**Salinee Jantrapirom, Luca Lo Piccolo, Dumnoensun Pruksakorn, Saranyapin Potikanond and Wutigri Nimlamool**

**Table S1.** The Cancer Genome Atlas (TCGA) and non-TCGA studies for breast, lung, and ovary cancer were screened for amplification and/or deletions of *UBQLNs*. Only non-redundant studies were included in this analysis. The database stores 3116, 884, and 583 samples of breast, lung, and ovary cancer, respectively.

| Cancer | Genetic Variants | |
|---|---|---|
| | Amplification | Deletion |
| Breast | 8/3116 (0.26%) (UBQLN1) | 0/3116 (0.0%) (UBQLN1) |
| | 15/3116 (0.48%) (UBQLN2) | 1/3116 (0.03%) (UBQLN2) |
| | 20/3116 (0.68%) (UBQLN3) | 15/3116 (0.48%) (UBQLN3) |
| | 446/3116 (14.96%) (UBQLN4) | 0/3116 (0.0% (UBQLN4) |
| Lung | 2/2884 (0.07%) (UBQLN1) | 15/2884 (0.52%) (UBQLN1) |
| | 12/2884 (0.42%) (UBQLN2) | 19/2884 (0.66%) (UBQLN2) |
| | 2/2884 (0.07%) (UBQLN3) | 12/2884 (0.42%) (UBQLN3) |
| | 171/2884 (5.39%) (UBQLN4) | 0/2884 (0.0%) (UBQLN4) |
| Ovary | 0/583 (0.0%) (UBQLN1) | 0/583 (0.0%) (UBQLN1) |
| | 15/583 (2.57%) (UBQLN2) | 3/583 (0.51%) (UBLQN2) |
| | 2/583 (0.34%) (UBLQN3) | 2/583 (0.34%) (UBQLN3) |
| | 59/583 (10.12%) (UBQLN4) | 0/583 (0.0%) (UBQLN4) |

**Table S2.** The Cancer Genome Atlas (TCGA) and non-TCGA studies for breast were analyzed in order to identify *UBLQN*'s mutations. Allele frequency is shown as percentage (%) of variants reads out of 103 total from Whole-exome sequencing of 817 breast invasive carcinoma tumor/normal pairs. Table lists all mutations with allele frequency higher than 15%.

| Gene | Mutation Type | Protein Change | Allele Frequency (%) |
|---|---|---|---|
| *UBLQN1* | Missense | Q539E | 47 |
| | Nonsense | Q176* | 31 |
| *UBQLN2* | Missense | V191G | 26 |
| | Missense | E42Q | 3 |
| *UBLQN3* | Missense | P108S | 26 |
| | Missense | I403L | 15 |
| *UBQLN4* | Missense | P108H | 30 |
| | Missense | Q422E | 36 |
| | Missense | M537T | 32 |
| | Missense | E273Q | 19 |
| | Missense | W333C | 17 |
| | Missense | S408T | 16 |

**Table S3.** The Cancer Genome Atlas (TCGA) and non-TCGA studies on Lung adenocarcinoma were analyzed in order to identify *UBLQN*'s mutations. Allele frequency is shown as percentage (%) of variants reads out of 150 total from Whole-exome sequencing of 660 lung ADC and 484 lung SqCC tumor/normal pairs. Table lists all mutations with allele frequency higher than 15%.

| Gene | Mutation Type | Protein Change | Allele Frequency (%) |
|---|---|---|---|
| *UBLQN1* | Nonsense | G499* | 53 |
| | Missense | L91W | 46 |
| | Frame_shift | T475Nfs*27 | 46 |
| | Missense | N577S | 41 |
| | Missense | R274C | 3 |
| | Missense | M454V | 27 |
| | Missense | G492E | 27 |
| | Missense | M235I | 17 |
| | Missense | L149I | 19 |
| | Missense | S333L | 16 |
| *UBQLN2* | Missense | P440Q | 68 |
| | Missense | H90N | 54 |
| | Missense | G481V | 54 |
| | Missense | G526C | 50 |
| | Missense | P414R | 47 |
| | Missense | V505F | 43 |
| | Missense | M271I | 40 |
| | Missense | M386I | 38 |
| | Missense | G288W | 30 |
| | Missense | Q173L | 23 |
| | Missense | S444L | 23 |
| | Missense | Q170H | 22 |
| | Missense | Q174P | 21 |
| | Missense | Q327H | 20 |
| | Missense | S140C | 17 |
| | Missense | S256R | 16 |
| *UBQLN3* | Missense | M546I | 88 |
| | Missense | I247L | 79 |
| | Missense | A268S | 71 |
| | Missense | G312A | 7 |
| | Missense | S294L | 51 |
| | Frame shift | P382Hfs*20 | 54 |
| | Missense | Q198H | 50 |
| | Missense | D321N | 48 |
| | Missense | Y258C | 48 |
| | Missense | R295S | 46 |
| | Missense | P214T | 44 |
| | Missense | L89Q | 41 |
| | Missense | E232D | 37 |
| | Missense | R53H | 34 |
| | Missense | R225L | 31 |
| | Missense | V240L | 31 |
| | Missense | A430S | 31 |
| | Missense | Q198K | 30 |
| | Missense | G431W | 30 |
| | Missense | Q636L | 30 |
| | Missense | A7S | 29 |
| | Missense | S106C | 29 |
| | Missense | P472T | 29 |
| | Missense | L566F | 29 |

| | | | |
|---|---|---|---|
| | Missense | D443Y | 28 |
| | Missense | M626G | 28 |
| | Missense | D263H | 27 |
| | Missense | M262I | 25 |
| | Missense | L511F | 25 |
| | Missense | G589V | 25 |
| | Missense | D284Y | 23 |
| | Missense | E101K | 21 |
| | Missense | S36T | 18 |
| | Missense | Q394K | 16 |
| | Missense | A516T | 16 |
| *UBQLN4* | Missense | P255A | 36 |
| | Missense | M261V | 30 |
| | Missense | M218I | 30 |
| | Missense | S516F | 27 |
| | Missense | V78D | 18 |

© 2020 by the authors. Licensee MDPI, Basel, Switzerland. This article is an open access article distributed under the terms and conditions of the Creative Commons Attribution (CC BY) license (http://creativecommons.org/licenses/by/4.0/).
